# SUMOylation Connects Cell Stress Responses and Inflammatory Control: Lessons From the Gut as a Model Organ

**DOI:** 10.3389/fimmu.2021.646633

**Published:** 2021-02-19

**Authors:** Jörn Karhausen, Luis Ulloa, Wei Yang

**Affiliations:** ^1^Department of Anesthesiology, Center for Perioperative Organ Protection, Duke University Medical Center, Durham, NC, United States; ^2^Department of Pathology, Duke University Medical Center, Durham, NC, United States

**Keywords:** small ubiquitin like modifier, post-translational modification, cell stress response, adaptive response mechanism, intestinal pathologies

## Abstract

Conjugation with the small ubiquitin-like modifier (SUMO) constitutes a key post-translational modification regulating the stability, activity, and subcellular localization of its target proteins. However, the vast numbers of identified SUMO substrates obscure a clear view on the function of SUMOylation in health and disease. This article presents a comprehensive review on the physiological relevance of SUMOylation by discussing how global SUMOylation levels—rather than specific protein SUMOylation—shapes the immune response. In particular, we highlight the growing body of work on SUMOylation in intestinal pathologies, because of the unique metabolic, infectious, and inflammatory challenges of this organ. Recent studies show that global SUMOylation can help restrain detrimental inflammation while maintaining immune defenses and tissue integrity. These results warrant further efforts to develop new therapeutic tools and strategies to control SUMOylation in infectious and inflammatory disorders.

## Introduction

Post-translational modifications (PTMs) form a crucial layer of regulation that substantially increases the functional repertoire of the existing proteome. One critical example is small ubiquitin-like modifier (SUMO) modification (SUMOylation), in which SUMO is covalently, but reversibly, linked to the lysine residues of target proteins. Because SUMOylation is highly responsive to endogenous and environmental stressors and because a large number of SUMO targets are transcription factors or nuclear proteins, this PTM is increasingly recognized as a key regulator in health and disease (1–3). Current literature on SUMOylation remains confusing, as pathways can be SUMOylated at multiple sites with seemingly conflicting consequences for its activity. What is striking, however, is that, following cell stress, SUMOylation rapidly increases across a broad set of target proteins and effectively re-programs cellular responses. This review summarizes the emerging knowledge of how this global SUMOylation response helps maintain cellular and tissue integrity by preventing exaggerated inflammation.

## The SUMO Pathway

Mammalian cells express 4 SUMO isoforms: SUMO1, SUMO2, SUMO3, and a less-studied SUMO4 [for detailed review of the SUMO pathway refer to (1, 4)]. Whereas, SUMO1 shares about 50% homology with SUMO2 and SUMO3, these last two isoforms are typically referenced together as SUMO2/3 because their close sequence homology does not allow distinction with currently available antibodies. Extensive redundancies between the SUMO isoforms have hindered defining their specific functions, but an essential role for SUMO2 has emerged from global deletion of *Sumo2*, which is embryonically lethal (5). By contrast, *Sumo1* and *Sumo3* null mice have no obvious phenotype (5, 6), and SUMO3 expression is significantly lower than SUMO2 in most tissues.

SUMO conjugation modulates protein activity, function, stability, subcellular localization, and interaction with other proteins. Similar to ubiquitination, SUMOylation comprises 4 enzymatic steps: The (1) maturation (through endopeptidase activity of the SUMO/sentrin specific peptidases [SENPs]), (2) activation (by forming an intermediate with the SUMO E1 activating enzyme SAE1/2), (3) conjugation (the ubiquitin-conjugating enzyme 9 [Ubc9] links SUMO to a lysine residue on the protein substrate), and (4) ligation (an optional step by which E3 ligases increase conjugation efficiency or specificity). Importantly, SUMOylation is extremely dynamic due to rapid de-SUMOylation mediated largely by the SENP family of isopeptidases (SENP1-3 and SENP5-8 in humans) (7).

Notably, technical advances have allowed large-scale, system-wide SUMO proteomics analyses (8, 9). A comprehensive analysis of 22 SUMO proteomic studies using human cells identified more than 3,000 SUMO targets with a large portion of these being transcriptional factors and chromatin-associated proteins, linked to accessing genetic information (9).

## SUMOylation Connects Cell Stress to Major Inflammatory Pathways

Various works have outlined SUMOylation as an important stress response conserved through evolution (10–13). These studies established that SUMOylation orchestrates cellular responses to heat shock, DNA damage, and mitochondrial-, osmotic-, oxidative-, hypoxic-, and ethanol stress (12–16).

Inflammation is a primary response to stress. While allowing for resolving infection and removing cellular debris, exaggerated inflammation directly threatens tissue integrity. Recent studies reveal a critical role of SUMOylation in both innate and adaptive immunity and provide a link between cellular stress sensing and inflammatory responses (17). However, a fundamental problem is that SUMOylation modulates often multiple and contradictory decision points within key inflammatory pathways, which leads to an inconsistent understanding of its true physiologic role. For example, NF-κB pathway activity is inhibited by SUMOylation at multiple levels, i.e., by stabilizing IκB and maintaining NF-kB repression (18); by interfering with the binding of co-activator CBP (19, 20); by transrepression of inflammatory target genes (21–24); and by regulating the stability of early response gene products such as the Nuclear receptor NR4A1 (25). However, SUMOylation can also stimulate NF-κB through de-repression of the negative regulators TANK (26) and NEMO (27). As a consequence, modulating SUMOylation has yielded conflicting results regarding NF-kB activity and inflammatory outcomes. As such, the SUMO E3 ligase protein inhibitor of activated STATs (PIAS) can inhibit NF-κB activation in some models (19, 28–32), but can also activate NF-κB after genotoxic stress (33). SENP2, which is particularly responsive to genotoxic stimuli, efficiently de-SUMOylates NEMO and limits NF-κB-dependent cell survival responses (34, 35). Correspondingly, depletion of SENP6 potentiates NF-κB-mediated induction of proinflammatory genes after endotoxin exposure *in vitro* and *in vivo* (36); however, endothelial knock-out of SENP1 in aortic grafts achieves the opposite and blunts endothelial responses to TNFα or IL-1β (37).

Another example of seemingly conflicting SUMOylation effects was observed in the regulation of NLRP3 activity. Here, Barry et al. (38) demonstrated that SUMO2/3 modification of NLRP3 at multiple lysine residues inhibits NLRP3 activation, whereas, stimulation-dependent NLRP3 de-SUMOylation through SENP6 and SENP7 promotes NLRP3 activation. However, NLRP3 modification with SUMO1 at one of these sites induces opposite results and promotes inflammasome activation and IL-1β secretion, which is reversed by de-SUMOylation with SENP3 (39).

## Changes in Global Sumoylation Levels Reprogram Inflammatory Responses

As outlined above, effects of SUMOylation on individual target proteins and pathways are complex and likely highly context-dependent. However, a striking observation under cell-stress conditions is the rapid net increase of SUMOylated proteins (16, 40–42). For the SUMO2/3 isoforms, this increase is readily appreciated by the detection of a high-molecular “smear”—a broad signal representing the large variety of SUMOylated proteins of different sizes—in Western blots (and the parallel decrease of un-bound SUMO2/3). A consistent body of evidence is emerging that identifies this increase of global SUMOylation as a broad-acting, adaptive response controlling inflammation. The consequences of rapid changes in cellular SUMOylation levels on inflammatory responses have not been comprehensively reviewed, and we will therefore summarize the available data produced by modifying global SUMOylation (predominantly by targeting the key E2-conjugase, Ubc9) and de-SUMOylation (by targeting different SENPs).

## Global Sumoylation and the Control of Immune Cell Functions

Deque et al. demonstrated that *Ubc9* null dendritic cells (DCs) responded to LPS with enhanced recruitment of RNA polymerase II to LPS-induced genes and consequently, with an exacerbated production of pro-inflammatory cytokines (43). Interestingly, this work also revealed that SUMOylation repressed LPS-induction of interferon-β (IFNβ1) and thus inhibited the crosstalk between type 1 IFN and pattern recognition receptor-ligand responses. In chimeric animals that received *Ubc9*-null bone marrow, these findings translated into an increased susceptibility to endotoxin shock, but resistance to viral infection, indicating that global SUMOylation effectively limits inflammation-induced pathology.

This anti-inflammatory role of SUMOylation is further supported by evolving evidence from studies using pharmacologic approaches. For example, the highly selective SUMOylation inhibitor TAK981—a new drug currently investigated as an adjuvant treatment of malignancies—prevents SUMOylation by inhibiting the transfer of SUMO to the E2 conjugating enzyme Ubc9 (44). In mouse bone-marrow and human peripheral blood mononuclear cell-derived DCs, TAK981 induces cell activation and maturation, triggers the production of inflammatory cytokines, and enhances priming and activation of antigen-reactive cytotoxic T cells. Notably, some of these effects were reversed by blocking interferon signaling (45). Conversely, we recently found that the synthetic organoselenium compound, ebselen, increases global SUMOylation levels by inhibiting SENP2 (46)—a protease with high catalytic activity for SUMO2/3 (47). Interestingly, ebselen has been shown to inhibit both DC-induced cytokine production by T cells and T cell-induced cytokine production by DCs (48).

Moreover, studies based on genetic modification of the SUMO pathway not only reveal the critical involvement of SUMOylation in the development and activity of lymphoid cells, mainly T cells, but also demonstrate its anti-inflammatory function. SENP1 is highly expressed at the early stages of T and B cell development and *Senp1*-null mice exhibit impairment specifically of T and B cell development (49). However, SUMOylation also modulates T cell activation by regulating T cell receptor (TCR)-signaling. TCR induces SUMO1 conjugation to control proximal (e.g., assembly of TCR with coreceptors) (50, 51) and distal [e.g., activation of Nuclear factor of activated T-cells (NFAT)] (52) signaling events, and mutation of the SUMOylation sites impairs cell activation and Th2 differentiation in primary CD4^+^ T cells and T cell lines. Along these lines, emerging data suggests that SUMO inhibition of T cells isolated from chronic lymphatic leukemia (CLL) patients, shifts the T cell balance toward Th1 polarization (53). Together, this could indicate that global SUMOylation is a critical determinant of the Th1/Th2 balance, which is further supported by a clear role of SUMOylation in supporting the number and functions of regulatory T cells (Treg), a specialized, inhibitory CD4^+^ T cell subtype. Here, pharmacologic inhibition of SUMOylation impairs Treg polarization in isolated CD4^+^ T cells (53), and Treg-specific *Ubc9* deletion impairs TCR-driven Treg proliferation and activation, and reproduces in animals the severe autoimmune phenotype seen with *Foxp3* deletion (54). Consistent with this, *Ubc9* deletion in macrophages attenuates the M2 (anti-inflammatory) program and reduces their capacity to induce Treg differentiation (55).

Studies of SUMOylation in other immune cells revealed that increased CD45 SUMOylation in *Senp1*-deficient mice promotes myeloid-derived suppressor cells (MDSC) immunosuppression function (56). Furthermore, siRNA knock-down of either SUMO1 or Ubc9 increases reactive oxygen species production from NADPH oxidases in neutrophils, whereas SUMO1 overexpression induces the opposite effect. This suggests that SUMOylation may control the ability of neutrophils to cause tissue injury or kill pathogens (57). Together, we have highlighted the diverse inflammation-regulatory effects of global SUMOylation in specific immune cell populations. To better understand how changes of global SUMOylation levels affect tissue outcomes in inflammation, we will next focus on studies that examined modulated SUMOylation levels in pre-clinical disease models.

## Global Sumoylation Controls Tissue Inflammation: Lessons From the Gut as a Model Organ

Parenchymal cells react to injurious stimuli with a complex and often interrelated set of inflammatory and adaptive responses. In balancing the needs of pathogen and cell debris removal (inflammation) and preservation of cellular function under adverse conditions (adaption), control of the immune environment is essential. This holds especially true for the gut, where the intestinal epithelium forms a single barrier between trillions of bacteria and an enormous mass of immune cells harbored within the intestinal walls. Because adverse environmental conditions constantly threaten epithelial integrity (58), adaptive responses are particularly well-developed in the gut (59–61). Indeed, the work of Demarque et al. impressively showed in inducible *Ubc9*-knockout mice that SUMOylation is crucial to intestinal maintenance through ensuring organized cell-renewal and differentiation, and by controlling mechanical stability of the epithelial monolayer (62). Together, this highlights the intestine as a model organ to study how SUMOylation regulates inflammation in an environment particularly challenged by metabolic, inflammatory, and infectious stressors.

### SUMO and Metabolic Stress

Epithelial functions generate substantial metabolic demands (63). Together with a vascular supply prone to shunting oxygen-rich blood away from the villus tip, this renders the intestinal epithelium particularly sensitive to reductions in blood flow and resultant ischemia/hypoxia (59). Interestingly, we found that SUMO2/3-conjugation, while highly responsive to perfusion abnormalities (64–66), did not follow the crypt-to-villus oxygen gradient (59), nor the matching expression of hypoxia-adaptive responses such as HIF-1α (67), but was restricted to villus crypt epithelia. However, intestinal ischemia/reperfusion (I/R) caused the rapid expansion of SUMO2/3 signal into villus tip epithelia establishing the stress-responsiveness of SUMO2/3 conjugation. This is an adaptive response, as demonstrated in *Ubc9* transgenic animals. In these animals, increased SUMOylation had a major effect on transcriptional responses regulating inflammatory cell recruitment pathways. Consistent with the dramatic reduction of neutrophil influx and the improved preservation of intestinal architecture in *Ubc9* transgenic animals after I/R, we found that pathways regulating inflammatory cell adhesion, tissue integrity and production of chemotactic factors were broadly modified in both whole tissue samples and in epithelia (42). Of note, compensatory overexpression of SUMO2/3 isoform in *Sumo1* null mice led to a comparable protective phenotype as observed in Ubc9 transgenic animals. Together, our data identify SUMOylation as a powerful mechanism by which the intestine controls the inflammatory environment during metabolic stress and highlights the particular importance of the SUMO2/3 isoforms in stress-adaptive, anti-inflammatory protection.

### SUMO in Inflammatory Bowel Diseases (IBD)

The noted prominent regulation of inflammatory responses raises the question of the role of SUMOylation in primary inflammatory diseases. Indeed, metabolic stress and dysregulated inflammation are key features of IBD (59, 68) and create conditions known to strongly induce SUMO2/3 conjugation (40, 41). Transcriptional analysis from *Ubc9* transgenic mice after I/R revealed a broad suppression of chemotactic factor expression, with many of them implicated in IBD pathogenesis [CXCL9 (69), CXCL16 (70), CCL20 (71), II17A (72), IL27 (73)]. For example, IL17A is a cytokine that can amplify inflammation by stimulating production of inflammatory mediators and thus promotes the recruitment of neutrophils and monocytes (74). IL17A has been implicated in many inflammatory diseases, including IBD (75). Singh et al. recently demonstrated that SUMOylation of ROR-γt—a key transcriptional regulator of IL17A—represses IL17A transcription (76). As a consequence, mice receiving Th17 cells expressing a SUMOylation-deficient mutant of ROR-γt in an adoptive transfer colitis model had significantly worse disease outcome measures compared to mice receiving Th17 cell expressing wild-type ROR-γt.

Surprisingly, while inflammatory processes such as rheumatoid arthritis (77–79) or I/R increase SUMOylation levels (42, 65, 80), Mustfa et al. reported the downregulation of Ubc9 and, with it, decreased SUMO-conjugation levels in the gut of murine and human IBD (81). This unexpected finding needs to be further confirmed in the context of disease stages and cell populations. Nonetheless, consistent with an anti-inflammatory function of SUMOylation, RNAi- knockdown of Ubc9 in cultured human epithelial cells significantly altered inflammatory gene expression, including that of key pro-inflammatory regulators RelA, cFos, and cJun. Furthermore, the level of Ubc9 downregulation correlated in both mouse and clinical samples with disease severity and the tissue expression of inflammatory cytokines (81). Following this logic, the same group developed a nanogel DNA delivery system to induce intestinal SUMOylation by enhancing expression of the E3 ligase, PIAS1 (protein inhibitor of activated STAT1) (82). These studies together support that increasing tissue SUMOylation blunts inflammation and tissue disruption in the gut.

### SUMO and Pathogen Responses

Growing evidence indicates that SUMOylation levels define the balance between destructive inflammation and effective defenses against pathogens within the gut. For example, *Shigella flexneri*, the etiological agent of bacterial dysentery, attacks colonic epithelia and causes massive inflammation-induced damage. Notably, mice haploinsufficient for *Ubc9*, display a hyper-invasive and hyper-inflammatory phenotype upon *in vivo* infection, emphasizing the importance of SUMOylation in the maintenance of intestinal permeability and mucosal inflammation (83). SILAC-based proteomics analysis revealed that invasive (vs. non-invasive) Shigella infection generally caused a reduction of SUMO2 modification. This affected a defined functional network of transcriptional regulators, where Shigella-induced changes in SUMOylation of regulators such as c-FOS, PPARc, and RXRa (24, 84, 85) were predicted to favor inflammation.

In line with this, SUMOylation is emerging as a key modulator of multiple host-pathogen interactions, with various pathogens actively targeting SUMOylation to their advantage. As such, *Listeria monocytogenes, Clostridium perfringens*, and *Streptococcus pneumoniae* induce proteasome-independent degradation of Ubc9 through closely related virulence factors (86), while *Shigella flexneri* targets the E1 ligase UBE2/SAE2 via proteasomal degradation (87), and S*almonella Typhimurium* targets Ubc9 via miRNA-mediated down-regulation (88). This attention given by bacterial (89), viral (90), and fungal (91) pathogens to the SUMO pathway highlights the critical role of this pathway in ensuring a balanced inflammatory response.

## Perspectives

**Figure 1:**
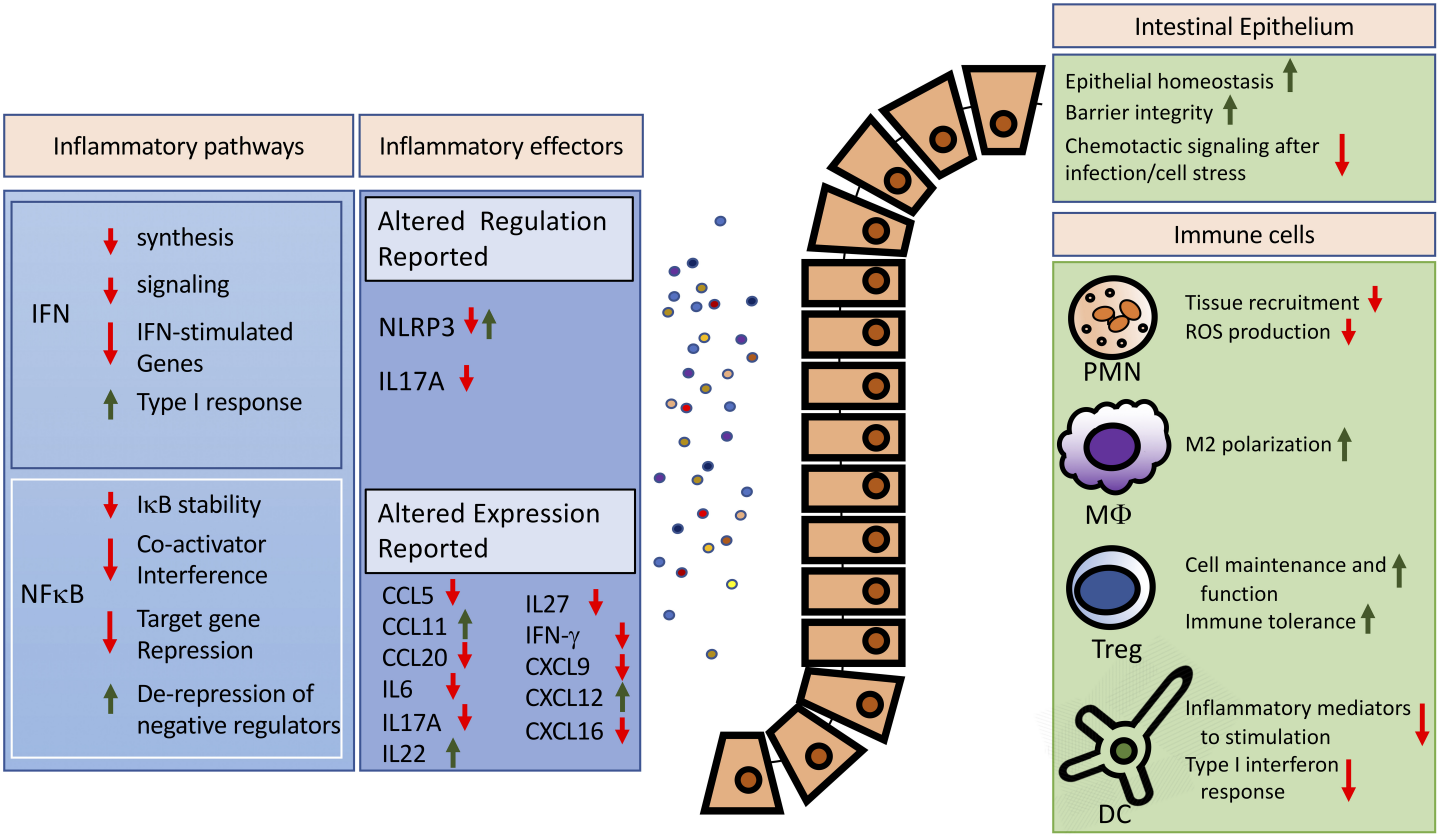
Potential anti-inflammatory effects of increased global SUMOylation in the intestine. Reported effects of SUMOylation on major inflammatory pathways and effectors are summarized on the left. For detailed reviews on SUMO-dependent regulation of the interferon or the NFκB pathway, we refer to excellent recent works (17, 92). Modulated expression of inflammatory mediators was reported in the intestines (42, 83). The right panel highlights cellular targets of SUMOylation and the potential effects of increased global SUMOylation in controlling exaggerated inflammation. IFN, interferon; PMN, polymorphonuclear leukocytes; MΦ, Macrophage; Treg, regulatory T cell; DC, dendritic cell.

In summary, mounting evidence supports global SUMOylation as a crucial cell stress response regulating inflammation. However, the appraisal of specific connections within this SUMO interactome remains complex, as SUMOylation of multiple components within a single pathway can produce contradictory effects. While this may serve to fine-tune specific responses in certain settings, it leaves unclear what is the actual impact of SUMOylation in diseases. Using the growing body of evidence from the gut as a model organ of particular metabolic, inflammatory, and infectious challenges (Figure 1), we further establish the notion that the global increase of SUMOylation during cellular stress constitutes a coordinated response to limit inflammation and preserve cellular and tissue integrity.

Many aspects of this response are still unclear. First and foremost, whether global increase of SUMOylation after cell stress is the equivalent of a flooding of the system with SUMO modifications, or rather the wide-sweeping but targeted introduction of a specific set of protein modifications. In line with this question, it remains unknown how SUMOylation itself is regulated following cell stress. The speed of the response (minutes *in vitro*) suggests a predominantly post-translational regulation of SUMO pathway components. Indeed, hypoxia-stimulated SUMOylation was not linked to increased expression of SUMO1, SUMO2, or SUMO3 by proteome or mRNA analysis but rather to the reversible inhibition of the catalytic activity, particularly of SENP1 and SENP3 (16). Yet overall, the decision points that trigger the increase of SUMOylation levels on such a grand scale remain to be determined.

Another fundamental consideration is the specific role of SUMO1 vs. SUMO2/3 in regulating inflammation during cell stress. Initial SUMO research focused on SUMO1 conjugation, yet growing evidence highlights the quick response of SUMO2/3 conjugation to cellular stressors, and the distinct roles of the SUMO isoforms. For example, NLRP3 is differentially regulated by SUMO1 vs. SUMO2/3 as discussed above (38, 39), while non-canonical type I interferon responses appear to be regulated by SUMO2/3, but not by SUMO1 (93). Similarly, our studies of SUMOylation in the gut also reveals that this process may play a distinct role in different cell populations (42). Consequently, studies of whole tissues, particularly within complex organs such as the gut, may yield conflicting results on how SUMOylation changes and affects inflammation. These results suggest a highly nuanced SUMO regulation, and future research will need to better determine isoform-specific and cell type-specific effects. A recently developed *Sumo2* conditional knockout mouse strain could be an invaluable tool (94).

Ultimately, to harness the beneficial potential of global SUMOylation, e.g., in autoimmune diseases, new pharmacologic interventions are needed. Here, oncology research has provided us with a number of specific SUMO inhibitors (95), but similar efforts are now needed to identify effective SUMO activators (46). Such studies can build on the presented evidence in multiple inflammatory disorders including I/R (42), IBD (82), and infectious disorders (83), which identify SUMOylation as a therapeutic target to restrain detrimental inflammation while maintaining immune defenses.

## Author Contributions

JK and WY developed the concept. All authors wrote and edited the final version of the manuscript.

## Conflict of Interest

The authors declare that the research was conducted in the absence of any commercial or financial relationships that could be construed as a potential conflict of interest.

## References

[1] Celen AB Sahin U. Sumoylation on its 25th anniversary: mechanisms, pathology, and emerging concepts. FEBS J. (2020) 287:3110–40. 10.1111/febs.15319 32255256

[2] Seeler JS Dejean A. Sumo and the robustness of cancer. Nat Rev Cancer. (2017) 17:184–97. 10.1038/nrc.2016.143 28134258

[3] Bernstock JD Yang W Ye DG Shen Y Pluchino S Lee YJ. Sumoylation in brain ischemia: patterns, targets, and translational implications. J Cereb Blood Flow Metab. (2018) 38:5–16. 10.1177/0271678X17742260 29148315 PMC5757445

[4] Chang HM Yeh ETH. Sumo: from bench to bedside. Physiol Rev. (2020) 100:1599–619. 10.1152/physrev.00025.2019 32666886 PMC7717128

[5] Wang L Wansleeben C Zhao S Miao P Paschen W Yang W. Sumo2 is essential while sumo3 is dispensable for mouse embryonic development. EMBO Reports. (2014) 15:878–85. 10.15252/embr.201438534 24891386 PMC4197045

[6] Zhang FP Mikkonen L Toppari J Palvimo JJ Thesleff I Janne OA. Sumo-1 function is dispensable in normal mouse development. Mol Cell Biol. (2008) 28:5381–90. 10.1128/MCB.00651-08 18573887 PMC2519746

[7] Hickey CM Wilson NR Hochstrasser M. Function and regulation of sumo proteases. Nat Rev Mol Cell Biol. (2012) 13:755–66. 10.1038/nrm3478 23175280 PMC3668692

[8] Yang W Paschen W. Sumo proteomics to decipher the sumo-modified proteome regulated by various diseases. Proteomics. (2015) 15:1181–91. 10.1002/pmic.201400298 25236368 PMC4382800

[9] Hendriks IA Vertegaal AC. A comprehensive compilation of sumo proteomics. Nat Rev Mol Cell Biol. (2016) 17:581–95. 10.1038/nrm.2016.81 27435506

[10] Zhou W Ryan JJ Zhou H. Global analyses of sumoylated proteins in saccharomyces cerevisiae. Induction of protein sumoylation by cellular stresses. J Biol Chem. (2004) 279:32262–8. 10.1074/jbc.M404173200 15166219 PMC2810850

[11] Augustine RC Vierstra RD. Sumoylation: re-wiring the plant nucleus during stress and development. Curr Opin Plant Biol. (2018) 45:143–54. 10.1016/j.pbi.2018.06.006 30014889

[12] Enserink JM. Sumo and the cellular stress response. Cell Div. (2015) 10:4. 10.1186/s13008-015-0010-1 26101541 PMC4476178

[13] Ryu HY Ahn SH Hochstrasser M. Sumo and cellular adaptive mechanisms. Exp Mol Med. (2020) 52:931–9. 10.1038/s12276-020-0457-2 32591648 PMC7338444

[14] He J Cheng J Wang T. Sumoylation-mediated response to mitochondrial stress. Int J Mol Sci. (2020) 21:5657. 10.3390/ijms21165657 PMC7460625 32781782

[15] Niskanen EA Palvimo JJ. Chromatin sumoylation in heat stress: to protect, pause and organise? Sumo stress response on chromatin. Bioessays. (2017) 39:263. 10.1002/bies.201600263 28440894

[16] Kunz K Wagner K Mendler L Holper S Dehne N Muller S. Sumo signaling by hypoxic inactivation of sumo-specific isopeptidases. Cell Rep. (2016) 16:3075–86. 10.1016/j.celrep.2016.08.031 27626674

[17] Adorisio S Fierabracci A Muscari I Liberati AM Ayroldi E Migliorati G. Sumo proteins: guardians of immune system. J Autoimmun. (2017) 84:21–8. 10.1016/j.jaut.2017.09.001 28919255

[18] Desterro JM Rodriguez MS Hay RT. Sumo-1 modification of ikappabalpha inhibits nf-kappab activation. Mol Cell. (1998) 2:233–9. 10.1016/S1097-2765(00)80133-1 9734360

[19] Jang HD Yoon K Shin YJ Kim J Lee SY. Pias3 suppresses nf-kappab-mediated transcription by interacting with the p65/rela subunit. J Biol Chem. (2004) 279:24873–80. 10.1074/jbc.M313018200 15140884

[20] Liu Y Bridges R Wortham A Kulesz-Martin M. Nf-kappab repression by pias3 mediated rela sumoylation. PLoS ONE. (2012) 7:e37636. 10.1371/journal.pone.0037636 22649547 PMC3359287

[21] Jennewein C Kuhn AM Schmidt MV Meilladec-Jullig V von Knethen A Gonzalez FJ. Sumoylation of peroxisome proliferator-activated receptor gamma by apoptotic cells prevents lipopolysaccharide-induced ncor removal from kappab binding sites mediating transrepression of proinflammatory cytokines. J Immunol. (2008) 181:5646–52. 10.4049/jimmunol.181.8.5646 18832723 PMC2679654

[22] Huang W Ghisletti S Saijo K Gandhi M Aouadi M Tesz GJ. Coronin 2a mediates actin-dependent de-repression of inflammatory response genes. Nature. (2011) 470:414–8. 10.1038/nature09703 21331046 PMC3464905

[23] Ghisletti S Huang W Ogawa S Pascual G Lin ME Willson TM. Parallel sumoylation-dependent pathways mediate gene- and signal-specific transrepression by lxrs and ppargamma. Mol Cell. (2007) 25:57–70. 10.1016/j.molcel.2006.11.022 17218271 PMC1850387

[24] Pascual G Fong AL Ogawa S Gamliel A Li AC Perissi V. A sumoylation-dependent pathway mediates transrepression of inflammatory response genes by ppar-gamma. Nature. (2005) 437:759–63. 10.1038/nature03988 16127449 PMC1464798

[25] Zhang L Xie F Zhang J Dijke PT Zhou F. Sumo-triggered ubiquitination of nr4a1 controls macrophage cell death. Cell Death Differ. (2017) 24:1530–9. 10.1038/cdd.2017.29 28622293 PMC5563982

[26] Renner F Saul VV Pagenstecher A Wittwer T Schmitz ML. Inducible sumo modification of tank alleviates its repression of tlr7 signalling. EMBO Rep. (2011) 12:129–35. 10.1038/embor.2010.207 21212807 PMC3049432

[27] Huang TT Wuerzberger-Davis SM Wu ZH Miyamoto S. Sequential modification of nemo/ikkgamma by sumo-1 and ubiquitin mediates nf-kappab activation by genotoxic stress. Cell. (2003) 115:565–76. 10.1016/S0092-8674(03)00895-X 14651848

[28] Xie B Liu X Yang J Cheng J Gu J Xue S. Pias1 protects against myocardial ischemia-reperfusion injury by stimulating ppargamma sumoylation. BMC Cell Biol. (2018) 19:24. 10.1186/s12860-018-0176-x 30419807 PMC6233564

[29] Liu B Yang R Wong KA Getman C Stein N Teitell MA. Negative regulation of nf-kappab signaling by pias1. Mol Cell Biol. (2005) 25:1113–23. 10.1128/MCB.25.3.1113-1123.2005 15657437 PMC544018

[30] Zhang J Xu LG Han KJ Wei X Shu HB. Piasy represses trif-induced isre and nf-kappab activation but not apoptosis. FEBS Lett. (2004) 570:97–101. 10.1016/j.febslet.2004.05.081 15251447

[31] Tahk S Liu B Chernishof V Wong KA Wu H Shuai K. Control of specificity and magnitude of nf-kappa b and stat1-mediated gene activation through piasy and pias1 cooperation. Proc Natl Acad Sci USA. (2007) 104:11643–8. 10.1073/pnas.0701877104 17606919 PMC1913887

[32] Wang X Jiang J Lu Y Shi G Liu R Cao Y. Tab2, an important upstream adaptor of interleukin-1 signaling pathway, is subject to sumoylation. Mol Cell Biochem. (2014) 385:69–77. 10.1007/s11010-013-1815-3 24096733

[33] Mabb AM Wuerzberger-Davis SM Miyamoto S. Piasy mediates nemo sumoylation and nf-kappab activation in response to genotoxic stress. Nat Cell Biol. (2006) 8:986–93. 10.1038/ncb1458 16906147

[34] Chen XL Wang SF Liang XT Liang HX Wang TT Wu SQ. Senp2 exerts an antitumor effect on chronic lymphocytic leukemia cells through the inhibition of the notch and nfkappab signaling pathways. Int J Oncol. (2019) 54:455–66. 10.3892/ijo.2018.4635 30431078 PMC6317657

[35] Lee MH Mabb AM Gill GB Yeh ET Miyamoto S. Nf-kappab induction of the sumo protease senp2: a negative feedback loop to attenuate cell survival response to genotoxic stress. Mol Cell. (2011) 43:180–91. 10.1016/j.molcel.2011.06.017 21777808 PMC3172129

[36] Liu X Chen W Wang Q Li L Wang C. Negative regulation of tlr inflammatory signaling by the sumo-deconjugating enzyme senp6. PLoS Pathog. (2013) 9:e1003480. 10.1371/journal.ppat.1003480 23825957 PMC3694847

[37] Qiu C Wang Y Zhao H Qin L Shi Y Zhu X. The critical role of senp1-mediated gata2 desumoylation in promoting endothelial activation in graft arteriosclerosis. Nat Commun. (2017) 8:15426. 10.1038/ncomms15426 28569748 PMC5461500

[38] Barry R John SW Liccardi G Tenev T Jaco I Chen CH. Sumo-mediated regulation of nlrp3 modulates inflammasome activity. Nat Commun. (2018) 9:3001. 10.1038/s41467-018-05321-2 30069026 PMC6070540

[39] Shao L Liu Y Wang W Li A Wan P Liu W. Sumo1 sumoylates and senp3 desumoylates nlrp3 to orchestrate the inflammasome activation. FASEB J. (2020) 34:1497–515. 10.1096/fj.201901653R 31914638

[40] Yang W Sheng H Thompson JW Zhao S Wang L Miao P. Small ubiquitin-like modifier 3-modified proteome regulated by brain ischemia in novel small ubiquitin-like modifier transgenic mice: putative protective proteins/pathways. Stroke. (2014) 45:1115–22. 10.1161/STROKEAHA.113.004315 24569813 PMC3966925

[41] Saitoh H Hinchey J. Functional heterogeneity of small ubiquitin-related protein modifiers sumo-1 versus sumo-2/3. J Biol Chem. (2000) 275:6252–8. 10.1074/jbc.275.9.6252 10692421

[42] Karhausen J Bernstock JD Johnson KR Sheng HX Ma Q Shen YT. Ubc9 overexpression and sumo1 deficiency blunt inflammation after intestinal ischemia/reperfusion. Lab Invest. (2018) 98:799–813. 10.1038/s41374-018-0035-6 29472640 PMC6397426

[43] Decque A Joffre O Magalhaes JG Cossec JC Blecher-Gonen R Lapaquette P. Sumoylation coordinates the repression of inflammatory and anti-viral gene-expression programs during innate sensing. Nat Immunol. (2016) 17:140–9. 10.1038/ni.3342 26657003

[44] National Cancer Institute. Sumoylation Inhibitor TAK-981. Available online at: https://www.cancer.gov/publications/dictionaries/cancer-drug/def/subasumstat (accessed February 6, 2021).

[45] Khattar M Song K Grossman S Xega K He X Idamakanti N. Tak-981: A first in class sumo inhibitor in phase 1 trials that promotes dendritic cell activation, antigen-presentation, and t cell priming [abstract]. Cancer Res. (2019) 79:Abstract nr 3252. 10.1158/1538-7445.AM2019-3252

[46] Bernstock JD Ye D Smith JA Lee YJ Gessler FA Yasgar A. Quantitative high-throughput screening identifies cytoprotective molecules that enhance sumo conjugation via the inhibition of sumo-specific protease (senp)2. FASEB J. (2017) 32:1677–91. 10.1096/fj.201700711R 29146736 PMC5892725

[47] Mendes AV Grou CP Azevedo JE Pinto MP. Evaluation of the activity and substrate specificity of the human senp family of sumo proteases. Biochimica et Biophysica Acta Mol Cell Res. (2016) 1863:139–47. 10.1016/j.bbamcr.2015.10.020 26522917

[48] Matsue H Edelbaum D Shalhevet D Mizumoto N Yang C Mummert ME. Generation and function of reactive oxygen species in dendritic cells during antigen presentation. J Immunol. (2003) 171:3010–8. 10.4049/jimmunol.171.6.3010 12960326

[49] Van Nguyen T Angkasekwinai P Dou H Lin FM Lu LS Cheng J. Sumo-specific protease 1 is critical for early lymphoid development through regulation of stat5 activation. Mol Cell. (2012) 45:210–21. 10.1016/j.molcel.2011.12.026 22284677 PMC3269036

[50] Wang XD Gong Y Chen ZL Gong BN Xie JJ Zhong CQ. Tcr-induced sumoylation of the kinase pkc-theta controls t cell synapse organization and t cell activation. Nat Immunol. (2015) 16:1195–203. 10.1038/ni.3259 26390157

[51] Wang QL Liang JQ Gong BN Xie JJ Yi YT Lan X. T cell receptor (tcr)-induced plc-gamma1 sumoylation via piasxbeta and pias3 sumo e3 ligases regulates the microcluster assembly and physiological function of plc-gamma1. Front Immunol. (2019) 10:314. 10.3389/fimmu.2019.00314 30873169 PMC6403162

[52] Xiong Y Yi Y Wang Y Yang N Rudd CE Liu H. Ubc9 interacts with and sumoylates the tcr adaptor slp-76 for nfat transcription in t cells. J Immunol. (2019) 203:3023–36. 10.4049/jimmunol.1900556 31666306

[53] Lam V Best SR Bruss N Liu T Hashiguchi Rowland T Huszar D. Pharmacologic inhibition of sumo-activating enzyme (sae) with tak-981 augments interferon signaling and regulates t cell differentiation in *ex vivo* studies of chronic lymphocytic leukemia (cll). Blood. (2019) 134:1760. 10.1182/blood-2019-127539

[54] Ding X Wang A Ma X Demarque M Jin W Xin H. Protein sumoylation is required for regulatory t cell expansion and function. Cell Rep. (2016) 16:1055–66. 10.1016/j.celrep.2016.06.056 27425617

[55] Wang F Sun F Luo J Yue T Chen L Zhou H. Loss of ubiquitin-conjugating enzyme e2 (ubc9) in macrophages exacerbates multiple low-dose streptozotocin-induced diabetes by attenuating m2 macrophage polarization. Cell Death Dis. (2019) 10:892. 10.1038/s41419-019-2130-z 31767832 PMC6877645

[56] Huang X Zuo Y Wang X Wu X Tan H Fan Q. Sumo-specific protease 1 is critical for myeloid-derived suppressor cell development and function. Cancer Res. (2019) 79:3891–902. 10.1158/0008-5472.CAN-18-3497 31186231

[57] Pandey D Chen F Patel A Wang CY Dimitropoulou C Patel VS. Sumo1 negatively regulates reactive oxygen species production from nadph oxidases. Arterioscler Thromb Vasc Biol. (2011) 31:1634–42. 10.1161/ATVBAHA.111.226621 21527745 PMC3464053

[58] Karhausen J Haase VH Colgan SP. Inflammatory hypoxia: role of hypoxia-inducible factor. Cell Cycle. (2005) 4:256–8. 10.4161/cc.4.2.1407 15655360

[59] Karhausen J Furuta GT Tomaszewski JE Johnson RS Colgan SP Haase VH. Epithelial hypoxia-inducible factor-1 is protective in murine experimental colitis. J Clin Invest. (2004) 114:1098–106. 10.1172/JCI200421086 15489957 PMC522241

[60] Kaser A Flak MB Tomczak MF Blumberg RS. The unfolded protein response and its role in intestinal homeostasis and inflammation. Exp Cell Res. (2011) 317:2772–9. 10.1016/j.yexcr.2011.07.008 21821022 PMC3392150

[61] Karin M Clevers H. Reparative inflammation takes charge of tissue regeneration. Nature. (2016) 529:307–15. 10.1038/nature17039 26791721 PMC5228603

[62] Demarque MD Nacerddine K Neyret-Kahn H Andrieux A Danenberg E Jouvion G. Sumoylation by ubc9 regulates the stem cell compartment and structure and function of the intestinal epithelium in mice. Gastroenterology. (2011) 140:286–96. 10.1053/j.gastro.2010.10.002 20951138

[63] Glover LE Lee JS Colgan SP. Oxygen metabolism and barrier regulation in the intestinal mucosa. J Clin Invest. (2016) 126:3680–8. 10.1172/JCI84429 27500494 PMC5096807

[64] Yang W Sheng H Warner DS Paschen W. Transient global cerebral ischemia induces a massive increase in protein sumoylation. J Cereb Blood Flow Metab. (2008) 28:269–79. 10.1038/sj.jcbfm.9600523 17565359

[65] Wang Z Wang R Sheng H Sheng SP Paschen W Yang W. Transient ischemia induces massive nuclear accumulation of sumo2/3-conjugated proteins in spinal cord neurons. Spinal Cord. (2013) 51:139–43. 10.1038/sc.2012.100 22945749

[66] Datwyler AL Lattig-Tunnemann G Yang W Paschen W Lee SL Dirnagl U. Sumo2/3 conjugation is an endogenous neuroprotective mechanism. J Cereb Blood Flow Metab. (2011) 31:2152–9. 10.1038/jcbfm.2011.112 21863037 PMC3210338

[67] Giles RH Lolkema MP Snijckers CM Belderbos M van der Groep P Mans DA. Interplay between vhl/hif1 alpha and wnt/beta-catenin pathways during colorectal tumorigenesis. Oncogene. (2006) 25:3065–70. 10.1038/sj.onc.1209330 16407833

[68] Lanis JM Kao DJ Alexeev EE Colgan SP. Tissue metabolism and the inflammatory bowel diseases. J Mol Med (Berl). (2017) 95:905–13. 10.1007/s00109-017-1544-2 28528514 PMC5696119

[69] Schroepf S Kappler R Brand S Prell C Lohse P Glas J. Strong overexpression of cxcr3 axis components in childhood inflammatory bowel disease. Inflamm Bowel Dis. (2010) 16:1882–90. 10.1002/ibd.21312 20848514

[70] Uza N Nakase H Yamamoto S Yoshino T Takeda Y Ueno S. Sr-psox/cxcl16 plays a critical role in the progression of colonic inflammation. Gut. (2011) 60:1494–505. 10.1136/gut.2010.221879 21471570

[71] Skovdahl HK Granlund A Ostvik AE Bruland T Bakke I Torp SH. Expression of ccl20 and its corresponding receptor ccr6 is enhanced in active inflammatory bowel disease, and tlr3 mediates ccl20 expression in colonic epithelial cells. PLoS ONE. (2015) 10:e0141710. 10.1371/journal.pone.0141710 26536229 PMC4633243

[72] Iboshi Y Nakamura K Fukaura K Iwasa T Ogino H Sumida Y. Increased il-17a/il-17f expression ratio represents the key mucosal t helper/regulatory cell-related gene signature paralleling disease activity in ulcerative colitis. J Gastroenterol. (2017) 52:315–26. 10.1007/s00535-016-1221-1 27178567

[73] Andrews C McLean MH Durum SK. Interleukin-27 as a novel therapy for inflammatory bowel disease: a critical review of the literature. Inflamm Bowel Dis. (2016) 22:2255–64. 10.1097/MIB.0000000000000818 27243591 PMC4992429

[74] Amatya N Garg AV Gaffen SL. Il-17 signaling: the yin and the yang. Trends Immunol. (2017) 38:310–22. 10.1016/j.it.2017.01.006 28254169 PMC5411326

[75] Patel DD Kuchroo VK. Th17 cell pathway in human immunity: lessons from genetics and therapeutic interventions. Immunity. (2015) 43:1040–51. 10.1016/j.immuni.2015.12.003 26682981

[76] Singh AK Khare P Obaid A Conlon KP Basrur V DePinho RA. Sumoylation of ror-gammat inhibits il-17 expression and inflammation via hdac2. Nat Commun. (2018) 9:4515. 10.1038/s41467-018-06924-5 30375383 PMC6207785

[77] Lao M Zhan Z Li N Xu S Shi M Zou Y. Role of small ubiquitin-like modifier proteins-1 (sumo-1) in regulating migration and invasion of fibroblast-like synoviocytes from patients with rheumatoid arthritis. Exp Cell Res. (2019) 375:52–61. 10.1016/j.yexcr.2018.12.011 30562482

[78] Wang C Xiao Y Lao M Wang J Xu S Li R. Increased sumo-activating enzyme sae1/uba2 promotes glycolysis and pathogenic behavior of rheumatoid fibroblast-like synoviocytes. JCI Insight. (2020) 5:135935. 10.1172/jci.insight.135935 32938830 PMC7526534

[79] Frank S Peters MA Wehmeyer C Strietholt S Koers-Wunrau C Bertrand J. Regulation of matrixmetalloproteinase-3 and matrixmetalloproteinase-13 by sumo-2/3 through the transcription factor nf-kappab. Ann Rheum Dis. (2013) 72:1874–81. 10.1136/annrheumdis-2012-202080 23417988

[80] Guo C Wei Q Su Y Dong Z. Sumoylation occurs in acute kidney injury and plays a cytoprotective role. Biochim Biophys Acta. (2015) 1852:482–9. 10.1016/j.bbadis.2014.12.013 25533125 PMC4386022

[81] Mustfa SA Singh M Suhail A Mohapatra G Verma S Chakravorty D. Sumoylation pathway alteration coupled with downregulation of sumo e2 enzyme at mucosal epithelium modulates inflammation in inflammatory bowel disease. Open Biol. (2017) 7:170024. 10.1098/rsob.170024 28659381 PMC5493774

[82] Yavvari PS Verma P Mustfa SA Pal S Kumar S Awasthi AK. A nanogel based oral gene delivery system targeting sumoylation machinery to combat gut inflammation. Nanoscale. (2019) 11:4970–86. 10.1039/C8NR09599J 30839018

[83] Fritah S Lhocine N Golebiowski F Mounier J Andrieux A Jouvion G. Sumoylation controls host anti-bacterial response to the gut invasive pathogen shigella flexneri. EMBO Rep. (2014) 15:965–72. 10.15252/embr.201338386 25097252 PMC4198040

[84] Bossis G Malnou CE Farras R Andermarcher E Hipskind R Rodriguez M. Down-regulation of c-fos/c-jun ap-1 dimer activity by sumoylation. Mol Cell Biol. (2005) 25:6964–79. 10.1128/MCB.25.16.6964-6979.2005 16055710 PMC1190241

[85] Choi SJ Chung SS Rho EJ Lee HW Lee MH Choi HS. Negative modulation of rxralpha transcriptional activity by small ubiquitin-related modifier (sumo) modification and its reversal by sumo-specific protease susp1. J Biol Chem. (2006) 281:30669–77. 10.1074/jbc.M604033200 16912044

[86] Ribet D Hamon M Gouin E Nahori MA Impens F Neyret-Kahn H. Listeria monocytogenes impairs sumoylation for efficient infection. Nature. (2010) 464:1192–5. 10.1038/nature08963 20414307 PMC3627292

[87] Lapaquette P Fritah S Lhocine N Andrieux A Nigro G Mounier J. Shigella entry unveils a calcium/calpain-dependent mechanism for inhibiting sumoylation. Elife. (2017) 6:27444. 10.7554/eLife.27444 29231810 PMC5745084

[88] Verma S Mohapatra G Ahmad SM Rana S Jain S Khalsa JK. Salmonella engages host micrornas to modulate sumoylation: a new arsenal for intracellular survival. Mol Cell Biol. (2015) 35:2932–46. 10.1128/MCB.00397-15 26100020 PMC4525320

[89] Srikanth CV Verma S. Sumoylation as an integral mechanism in bacterial infection and disease progression. Adv Exp Med Biol. (2017) 963:389–408. 10.1007/978-3-319-50044-7_22 28197924

[90] El Motiam A Vidal S Seoane R Bouzaher YH Gonzalez-Santamaria J Rivas C. Sumo and cytoplasmic rna viruses: from enemies to best friends. Adv Exp Med Biol. (2020) 1233:263–77. 10.1007/978-3-030-38266-7_11 32274761 PMC7144409

[91] Sahu MS Patra S Kumar K Kaur R. Sumoylation in human pathogenic fungi: role in physiology and virulence. J Fungi (Basel). (2020) 6:32. 10.3390/jof6010032 32143470 PMC7096222

[92] El-Asmi F McManus FP Thibault P Chelbi-Alix MK. Interferon, restriction factors and sumo pathways. Cytokine Growth Factor Rev. (2020) 55:37–47. 10.1016/j.cytogfr.2020.03.001 32591223

[93] Crowl JT Stetson DB. Sumo2 and sumo3 redundantly prevent a noncanonical type i interferon response. Proc Natl Acad Sci USA. (2018) 115:6798–803. 10.1073/pnas.1802114115 29891701 PMC6042150

[94] Yu S Galeffi F Rodriguiz RM Wang Z Shen Y Lyu J. Small ubiquitin-like modifier 2 (sumo2) is critical for memory processes in mice. FASEB J. (2020) 34:14750–67. 10.1096/fj.202000850RR 32910521 PMC7606628

[95] Yang Y Xia Z Wang X Zhao X Sheng Z Ye Y. Small-molecule inhibitors targeting protein sumoylation as novel anticancer compounds. Mol Pharmacol. (2018) 94:885–94. 10.1124/mol.118.112300 29784649

